# Management and optimisation in the preoperative phase for patients with a fractured hip

**DOI:** 10.1530/EOR-2026-0044

**Published:** 2026-05-01

**Authors:** Geert Meermans, Jeroen C van Egmond

**Affiliations:** Department of Orthopaedics, Bravis Hospital, Roosendaal, The Netherlands

**Keywords:** hip fracture, preoperative optimisation, orthogeriatric care, early surgery, multimodal analgesia, delirium prevention, multidisciplinary pathways, perioperative management

## Abstract

- Hip fractures represent a time-critical, systemic condition in older adults, characterised by frailty, multimorbidity, inflammation, immobility, pain, and high risk of medical complications and mortality.
- While population ageing is expected to drive a continued rise in absolute hip fracture numbers, contemporary evidence demonstrates declining age-standardised incidence in many high-income countries, highlighting the importance of optimising perioperative care pathways to mitigate morbidity and mortality.
- Contemporary evidence consistently supports early surgical management, typically within 24–48 h, provided reversible medical issues are addressed in parallel rather than through prolonged preoperative workup.
- Orthogeriatric co-management and structured multidisciplinary pathways reduce time to surgery, delirium incidence, length of stay, and mortality while improving functional recovery.
- Key optimisation domains include multimodal opioid-sparing analgesia, delirium prevention bundles, targeted cardiovascular stabilisation, pragmatic management of anti-thrombotic therapy, anaemia, infection, and individualised anaesthetic strategies.
- Evidence supports protocol-driven, multicomponent care bundles over isolated interventions to enable safe early surgery, reduce complications, and improve functional recovery in this vulnerable population.

## Introduction

**Figure 1:**
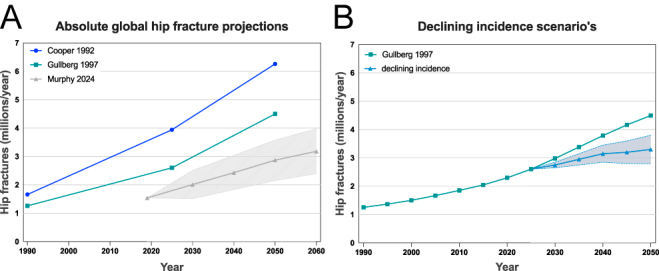
(A) Global hip fracture projections as reported by Cooper *et al.* (1) and Gullberg *et al.* (3). For the global hip fracture projection of Murphy *et al.* (4), the estimates are derived from England/Wales/Northern Ireland demographics using scenario-based scaling. The dotted lines represent the upper and lower bounds of high- and low-incidence settings. (B) Global hip fracture projection taking into account declining age-standardised hip fracture rates as reported in multiple high-income countries. The dotted lines represent the upper and lower bounds of high- and low-incidence settings.

Projections of hip fracture burden consistently anticipate a marked rise in absolute fracture numbers driven primarily by population ageing (Fig. 1A) (1, 2, 3, 4, 5, 6, 7). However, more recent epidemiological evidence has challenged the assumption of stable incidence. Multiple high-income countries have reported sustained declines in age-standardised hip fracture rates since the late 1990s, particularly among older adults, likely reflecting improvements in osteoporosis management, fall prevention, and overall health status (2, 8, 9, 10). While absolute fracture numbers are still expected to rise in most settings due to population ageing, the magnitude of this increase is highly sensitive to future trends in hip fracture incidence (Fig. 1B).

Hip fractures represent a sentinel event for older adults, frequently triggering rapid loss of independence, prolonged disability, and increased mortality. Contemporary perspectives increasingly frame hip fractures as a systemic disease rather than an isolated orthopaedic injury, reflecting the interplay of frailty, inflammation, immobilisation, pain, delirium, and decompensation of chronic disease (11, 12, 13). Hip fractures initiate a cascade of pain, bleeding, immobility, and systemic inflammatory, hypercoagulable, and catabolic responses that predispose patients to medical complications and early death (11). Timely surgical management is widely regarded as a cornerstone of optimal care for patients sustaining a hip fracture because delay to operative fixation or arthroplasty prolongs exposure to these harmful states, increases the duration of bed rest, and may exacerbate pre-existing comorbidities, thereby worsening outcomes (14, 15, 16, 17, 18, 19).

Current international guidelines reflect this balance of evidence, generally recommending surgery within 24–48 h of admission, provided there are no medical contraindications (20, 21). The precise definition of ‘early’ surgery remains debated, with thresholds ranging from 24 to 48 h across healthcare systems. Recent works have focused on whether even more accelerated pathways confer additional benefit. The HIP ATTACK trial found that patients who underwent surgery a median of 6 h versus 24 h after their hip fracture was diagnosed did not have a significantly lower risk of mortality or major complications (22). This supports the principle that hip fracture surgery should be performed as early as safely possible. While short delays may be justified to correct reversible medical issues, prolonged postponement is consistently associated with increased mortality and morbidity. The emphasis should, therefore, shift from debating exact time thresholds to developing organisational models that prioritise hip fracture surgery, enable rapid medical optimisation, and minimise avoidable delays (Table 1).

**Table 1:** Principles of care in patients with a hip fracture.

Rapid diagnosis
Timely surgery
Physiological stabilisation
Opioid-sparing analgesia
Medication optimisation
Delirium prevention
Geriatric-informed care
Early mobilisation
Nutrition optimisation
Discharge planning
Secondary fracture prevention

Therefore, a modern, evidence-aligned approach treats preoperative optimisation as a structured, parallel process designed to enable early surgery rather than an open-ended workup. This review summarises the scientific evidence and practical principles for preoperative management and optimisation in hip fracture patients, emphasising interventions supported by clinical trials, systematic reviews, and high-quality observational studies.

## Principles

Hip fractures in older adults represent a complex medical, surgical, and rehabilitative emergency rather than an isolated orthopaedic injury. Contemporary best practice, therefore, emphasises a multidisciplinary model of care (Table 2), in which orthopaedic surgeons, geriatricians or internists, anaesthetists, nurses, physiotherapists, occupational therapists, pharmacists, dieticians, and social workers collaborate within a structured clinical pathway. International guidance from bodies such as the National Institute for Health and Care Excellence and the American Academy of Orthopaedic Surgeons consistently frames hip fracture care as a coordinated system rather than a single operative (20, 21).

**Table 2:** Overview of multidisciplinary perioperative care for hip fracture patients.

Discipline	Key responsibilities preoperatively
Orthopaedics	Early surgery planning, fracture stabilisation
Geriatrics/internal medicine	Frailty, comorbidity, delirium prevention, medication optimisation
Anaesthesia	Risk stratification, analgesia strategy
Nursing	Hydration, orientation, mobilisation
Physiotherapy	Early mobilisation planning
Pharmacy	Medication reconciliation
Dietetics	Nutritional optimisation

A core principle of multidisciplinary care is parallel rather than sequential assessment. Early senior review by surgery, medicine, and anaesthesia allows rapid identification of reversible problems, such as hypovolaemia, anaemia, electrolyte disturbances, uncontrolled heart failure, arrhythmias, hypoxia, or sepsis, while avoiding unnecessary investigations that delay surgery. Multidisciplinary pathways are consistently associated with shorter time to theatre because optimisation occurs in real time rather than via prolonged medical clearance. Orthogeriatric co-management addresses these complexities by combining orthopaedic and geriatric expertise in a single pathway by shared care on an orthopaedic ward, geriatric-led fracture wards with orthopaedic input, or dedicated hip fracture units. This can lead to faster mobility, less delirium, shorter length of stay, and lower mortality (13, 23, 24). Orthogeriatric care is increasingly defined as a set of core components such as early comprehensive geriatric assessment, shared decision-making, delirium prevention, mobilisation, discharge planning, and secondary prevention, with consensus efforts proposing minimal and optimal standards to reduce variation (25). Finally, malnutrition is highly prevalent among older patients with hip fractures and significantly impacts recovery and survival; therefore, routine nutritional screening and early intervention is needed to improve outcomes and reduce healthcare burden (26).

## Management

### Pain

Poor pain control contributes to immobility, impaired respiratory effort, sleep disruption, stress responses (sympathetic activation, hypercoagulability, and catabolism), and delirium risk, while heavy reliance on systemic opioids increases the likelihood of adverse events such as sedation, respiratory depression, nausea, urinary retention, constipation, and delirium (27, 28, 29, 30). Preoperative analgesia strategies, therefore, aim to achieve rapid pain relief while minimising opioid exposure, enabling early mobilisation and nursing care, and facilitating neuraxial anaesthesia positioning (31, 32, 33). Multimodal systemic analgesia and peripheral nerve blocks (PNBs) have become central components of multimodal hip fracture pathways for this reason (34, 35, 36).

Preoperative multimodal pain management in hip fracture patients aims to improve analgesia while reducing reliance on systemic opioids. Multimodal regimens commonly include scheduled paracetamol or acetaminophen and non-steroidal anti-inflammatory drugs (NSAIDs) or COX-2 inhibitors, which have been recommended in hip fracture pain protocols as part of basic multimodal analgesia because they contribute to effective pain relief and opioid sparing when not contraindicated (37). Data suggest that implementing structured multimodal approaches in hip fracture populations is associated with lower postoperative pain scores and reduced opioid consumption, supporting their integration into clinical pathways (38, 39, 40). Although the optimal combination of agents remains the subject of ongoing research, consensus guidance emphasises preoperative use of non-opioid analgesics to improve comfort and facilitate mobilisation in this frail population.

High-quality evidence supports the claim that PNBs provide clinically meaningful early pain reduction (Table 3). In a Cochrane systematic review of adults with a hip fracture, PNBs used preoperatively, postoperatively, or as a supplement to general anaesthesia reduced pain on movement within 30 min of placement, the risk of acute confusional state, the risk of chest infection, and time to first mobilisation (41). Modern hip fracture pathways favour simple, safe, and motor-sparing regional techniques. In the past, femoral nerve block (42, 43) and 3-in-1 block were most commonly used and effective, but often limited by motor block and variable spread (44, 45). Fascia iliaca compartment block (FICB) emerged as a standard of practice (46, 47, 48, 49). Nowadays, there is a growing adoption of the motor-sparing pericapsular nerve group (PENG) block as part of multimodal analgesia pathways (Table 3) (50, 51, 52). However, two systematic reviews concluded that current evidence is insufficient to demonstrate the superiority of PENG compared with FICB or FNB in the perioperative management of hip fracture repair (53, 54). For maximal effect, nerve blocks should be delivered early, ideally in the emergency department or soon after admission, embedded within standardised analgesic and delirium prevention protocols, and paired with clear rescue analgesia plans and mobilisation safeguards.

**Table 3:** Peripheral nerve block techniques for analgesia in hip fracture: anatomical targets, clinical advantages, and limitations.

Technique	Main target/coverage	Pros	Cons/limitations
Femoral nerve block (FNB)	Femoral nerve (anterior hip/thigh)	Dense, reliable analgesia; rapid onset; straightforward with ultrasound	Quadriceps weakness → fall risk and may hinder early mobilisation; limited obturator/lateral cutaneous coverage
3-in-1 block	Intended femoral + lateral femoral cutaneous (LFC) + obturator (often femoral predominant in practice)	Historically used; single injection concept	Often inconsistent obturator/LFC coverage; largely supplanted by FICB or PENG
Lumbar plexus block	Lumbar plexus (femoral + obturator + LFC)	Very dense, broad analgesia; can reduce opioid requirements; continuous techniques can provide prolonged analgesia	Technically demanding; deeper block with higher complication risk (e.g. hematoma in anticoagulated patients, inadvertent epidural spread, hypotension); less suited to ED/rapid pathways
Fascia iliaca compartment block (FICB)	Femoral nerve + LFC; obturator coverage variable	Simple; can be delivered in ED/ward; generally safe (low vascular/neuraxial risk); scalable for pathways	Obturator block inconsistent → variable analgesia; can cause quadriceps weakness (less than femoral block but still possible); landmark technique less reliable than US
Pericapsular nerve group block (PENG)	Articular branches to anterior hip capsule (femoral/obturator/accessory obturator)	Often excellent hip-specific analgesia; typically motor-sparing	Requires ultrasound + training; may not cover skin/incision pain alone; comparative effectiveness vs FICB/FNB still evolving

### Delirium

Delirium is one of the most common and serious complications encountered in the care of patients with a hip fracture, particularly among older adults with frailty and multiple comorbidities. In hip fracture populations, reported incidence rates of delirium vary widely, typically ranging from 15% to more than 50%, depending on patient characteristics, diagnostic criteria, and timing of assessment (55, 56, 57). Prevalence is highest in the elderly, in those with pre-existing cognitive impairment or dementia, and in patients exposed to pain, infection, polypharmacy, or physiological stress before surgery (58, 59). Delays to surgery prolong pain, immobility, and exposure to the stressful hospital environment, all of which increase delirium incidence (60, 61). Numerous observational studies and meta-analyses have demonstrated that delirium is independently associated with increased mortality, prolonged hospital length of stay, higher rates of medical complications, poorer functional recovery, and increased likelihood of institutionalisation (55, 62, 63). Preventative measures for delirium can be broadly categorised into (1) non-pharmacological multicomponent interventions, (2) optimisation of pain management, (3) avoidance of high-risk medications, and (4) system-level models of care (Table 4).

**Table 4:** Core strategies for delirium prevention in patients with a hip fracture.

Category	Domain	Preventative measures
Non-pharmacological	Cognitive orientation	Clocks, calendars, frequent reorientation, family engagement
	Sleep preservation	Minimise night-time disturbances; avoid sedatives where possible
	Hydration and nutrition	Prompt IV/oral fluids; early nutrition; avoid prolonged fasting
	Early mobilisation	Sit out of bed and ambulate as early as safely possible
	Vision and hearing aids	Ensure glasses and hearing devices are available
Pain management	Pain control	Early multimodal analgesia; avoid untreated pain; minimise excessive opioids
Medication	Medication review	Avoid benzodiazepines, anticholinergics, and unnecessary psychoactive drugs
System-level	Multidisciplinary care	Coordinated medical, nursing, therapy, and pharmacy input
	Oxygenation	Monitor saturation; supplemental oxygen if hypoxic
	Infection prevention and early treatment	Prompt identification of pneumonia, UTI, sepsis
	Bladder and bowel care	Avoid urinary retention and constipation

Among the preventative measures, non-pharmacological multicomponent interventions have the strongest evidence base. Programs derived from the Hospital Elder Life Program (HELP) framework – which address orientation, sleep, hydration, nutrition, mobilisation, sensory impairment, and cognitive stimulation – have consistently demonstrated reductions in delirium incidence across medical and surgical populations (64). In hip fracture cohorts, comprehensive geriatric care models incorporating these principles are associated with lower delirium rates compared with usual orthopaedic care (12, 23). A Cochrane review examining interventions for preventing delirium in hospitalised non-ICU patients concluded that multicomponent non-pharmacological strategies significantly reduced delirium incidence, with moderate-certainty evidence (64).

Pain is a central and modifiable precipitant of delirium in hip fracture patients. Both untreated pain and excessive opioid use increase delirium risk, highlighting the need for balanced, multimodal analgesia. Observational studies have demonstrated higher delirium rates in patients with poorly controlled pain, independent of age and comorbidity (29). Regional analgesia, particularly PNBs, has therefore gained attention as a preventative strategy. Meta-analyses focusing on hip fracture populations suggest that PNBs may reduce delirium incidence, likely through improved pain control and opioid-sparing effects (41, 65). While not all studies demonstrate a statistically significant reduction, the overall direction of evidence supports regional analgesia as part of a delirium prevention bundle rather than as a stand-alone intervention.

Medication management is another critical component of delirium prevention. Older hip fracture patients are particularly vulnerable to the deliriogenic effects of benzodiazepines, anticholinergic drugs, sedative hypnotics, and high-dose opioids. Systematic reviews have consistently identified these medications as risk factors for delirium (66, 67). Preventative strategies, therefore, emphasise regular medication review, avoidance of unnecessary psychoactive drugs, cautious opioid dosing, and preference for non-opioid and regional analgesic techniques where feasible. Importantly, pharmacological prophylaxis with antipsychotics or other agents has not demonstrated consistent benefit and is not recommended for routine delirium prevention in hip fracture patients (68). Medication management is critical not only for delirium prevention, but also for preventing subsequent falls, since elderly patients frequently use multiple medications. These medications have several interactions and, sometimes, orthostatic consequences; therefore, a structured medication review by a geriatrician is a core component of comprehensive geriatric assessment and is associated with fewer adverse geriatric outcomes (69, 70, 71).

System-level models of care represent one of the most effective approaches to delirium prevention in hip fracture populations. Orthogeriatric care, defined by shared management between orthopaedic surgeons and geriatricians, has been associated with lower delirium incidence, shorter hospital stay, and improved functional outcomes compared with traditional models (12, 23). These benefits likely arise from early identification of high-risk patients, proactive management of medical issues, structured delirium screening, and implementation of multicomponent prevention strategies.

### Cardiovascular

Cardiovascular disease is highly prevalent in hip fracture populations ranging from 50 to 80% and represents a major contributor to perioperative morbidity and mortality (72, 73, 74, 75). Contemporary guidance emphasises rapid, targeted stabilisation aimed at facilitating early surgery rather than prolonged preoperative workup. The National Institute for Health and Care Excellence recommends immediate correction of reversible cardiovascular abnormalities – particularly uncontrolled heart failure, clinically significant arrhythmias, and active myocardial ischemia – while avoiding unnecessary investigations that delay operative treatment (20). Myocardial injury is frequently under-recognised in this cohort, with perioperative troponin elevation occurring in a substantial proportion of patients and independently predicting short- and long-term mortality after hip fracture surgery (76). These findings support early risk identification and enhanced monitoring rather than postponement of surgery.

Heart failure and atrial fibrillation are particularly important targets for optimisation. Pre-existing heart failure markedly increases postoperative complications and mortality, especially when perioperative decompensation occurs (77). Patients developing both pre- and postoperative heart failure experience the poorest survival, highlighting the importance of euvolaemia, correction of precipitating factors such as anaemia and infection, and continuation of essential heart failure therapies where feasible. Atrial fibrillation, whether chronic or perioperative, is similarly associated with increased one-year mortality following hip fracture repair (78). Management should, therefore, focus on treating reversible triggers, including pain, hypovolaemia, hypoxia, and electrolyte disturbance, alongside pragmatic rate control strategies. Anticoagulation decisions must be integrated into structured perioperative protocols to balance bleeding and thromboembolic risks (see the next section).

**Figure 2:**
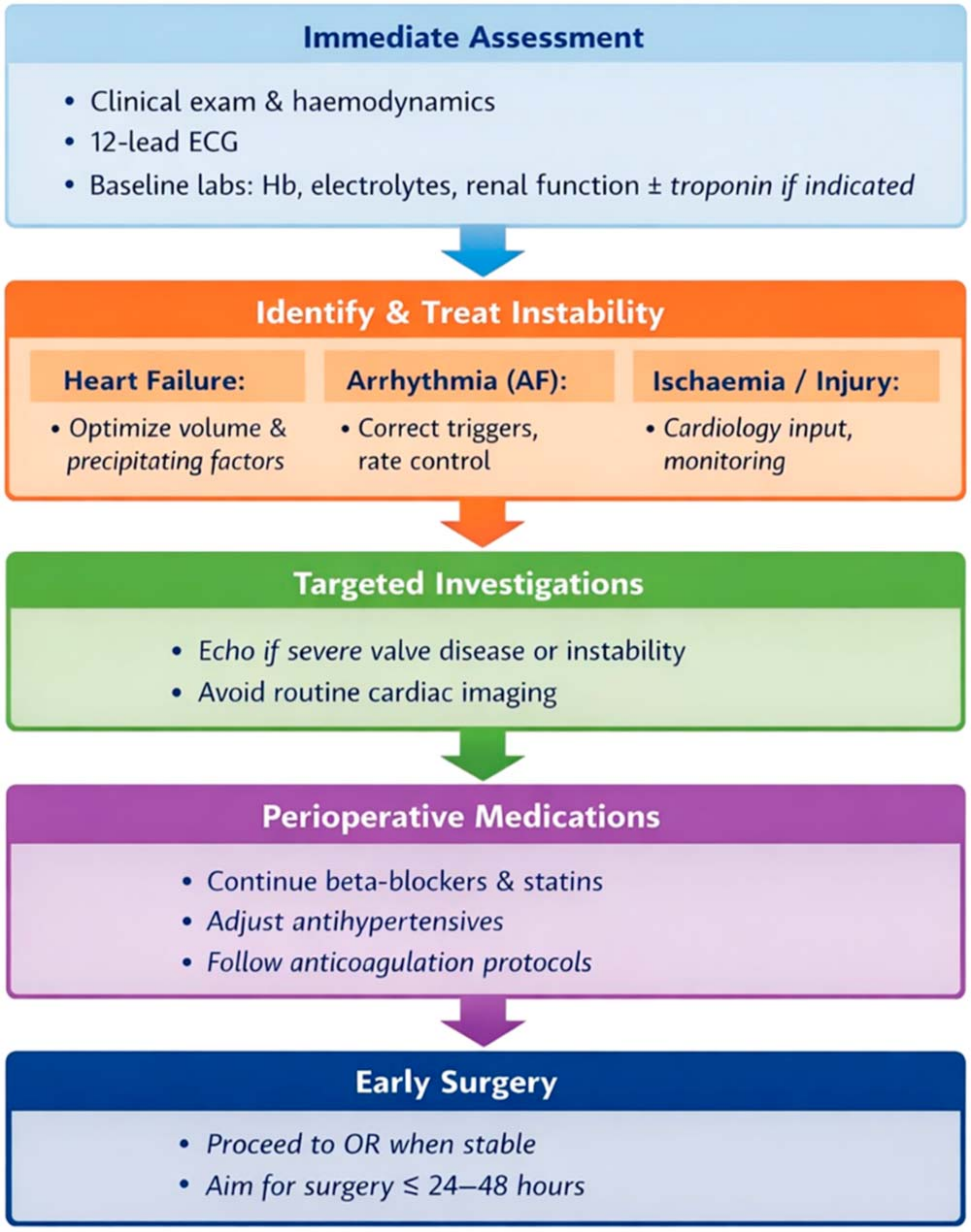
Cardiovascular optimisation in hip fracture patients.

The selective use of cardiac investigations is crucial to avoid avoidable surgical delay (Fig. 2). While transthoracic echocardiography is valuable when severe valvular diseases, new heart failure, or unexplained haemodynamic instability is suspected, routine preoperative echocardiography has repeatedly been shown to prolong time to surgery without clear outcome benefit (79, 80, 81). Hip fracture-specific anaesthesia guidance similarly cautions against indiscriminate cardiac testing, noting that delays associated with preoperative echocardiography are linked to worse outcomes (82). Focused clinical assessment with targeted imaging only when results are likely to alter immediate management is, therefore, recommended. Perioperative medication strategies should prioritise continuation of chronic beta-blockers and statins where appropriate, with cautious adjustment of antihypertensive therapy to avoid hypotension, consistent with broader perioperative cardiovascular guidelines (83).

Overall, cardiovascular optimisation in hip fracture patients is best achieved through rapid identification and correction of instability rather than exhaustive investigation. Evidence consistently demonstrates that heart failure, atrial fibrillation, and myocardial injury are strong predictors of adverse outcomes, while unnecessary diagnostic processes delay surgery and worsen prognosis. Embedding pragmatic cardiovascular assessment within multidisciplinary hip fracture pathways allows early surgery to proceed safely while minimising perioperative cardiac complications.

### Anti-thrombotic medication

Anti-thrombotic medication use is increasingly common among older adults who sustain a hip fracture and has become a major determinant of perioperative decision-making. The relevant drugs include oral anticoagulants, vitamin K antagonists (VKAs) such as warfarin and direct oral anticoagulants (DOACs), and antiplatelet agents (aspirin, clopidogrel, and dual antiplatelet therapy). In contemporary hip fracture cohorts, a meaningful minority of patients present while taking these agents. For example, a study of geriatric hip fracture patients reported that >6% were taking chronic warfarin on admission, consistent with earlier estimates of approximately 4–10% warfarin/VKA prevalence across settings and time periods (84, 85, 86). DOAC exposure has grown rapidly; multiple cohort studies report ∼10–20% DOAC prevalence in some modern series (with variability by country, age, and comorbidity burden) (87, 88, 89). Antiplatelet use is even more frequent; reviews and observational studies indicate that aspirin and/or clopidogrel are common in hip fracture populations, reflecting the high background prevalence of cardiovascular disease in the oldest age groups (90, 91). This epidemiology matters because anti-thrombotics affect (1) time to surgery, (2) bleeding and transfusion risk, (3) anaesthetic options, particularly neuraxial techniques, and (4) the competing risk of thrombotic events if agents are withheld inappropriately.

A clinically useful approach is, therefore, drug-specific and indication-specific: treat the hip fracture as urgent surgery, stratify the need for reversal and the urgency of surgery, and re-initiate therapy postoperatively when haemostasis is secure (Table 5). Vitamin K antagonist-treated hip fracture patients often face a delay because surgery is typically deferred until the INR is corrected. Several studies support standardised reversal pathways to reduce delays. For patients requiring faster normalisation, prothrombin complex concentrate (PCC) can reverse warfarin anticoagulation within hours and is frequently used when urgent surgery is needed (86, 92, 93, 94). DOAC management is more heterogeneous because drug effect depends on time since last dose, renal function, and the specific agent. However, the emerging direction of evidence is that routine surgical delay solely due to DOAC use is often unnecessary (95, 96, 97, 98). Pragmatic measures increasingly adopted in published protocols include documenting last DOAC dose, assessing renal function, and prioritising surgery once a minimum interval has passed often within the 24–48 h window where possible. For antiplatelet agents, the traditional impulse to delay surgery for several days to allow platelet function recovery has been increasingly challenged. Several reviews concluded that early surgery in patients taking clopidogrel and/or aspirin appears safe (90, 99, 100, 101, 102).

**Table 5:** Pre-operative management of blood-thinning medication in hip fracture surgery.

Drug class/examples	Preoperative assessment/monitoring	Practical preoperative plan for urgent hip fracture surgery	Reversal/bridging	When to restart
Antiplatelet – single agent (aspirin, clopidogrel, ticagrelor, or prasugrel)	Consider functional platelet testing if available (especially if neuraxial anaesthesia planned)	Do not routinely delay solely for single antiplatelet therapy; proceed with appropriate anaesthetic plan. (Elective/high-bleed-risk procedures: typical cessation windows exist, but these are often not feasible in hip fractures)	No routine reversal. Coordinate with anaesthesia/cardiology if high bleeding risk or neuraxial planned	Guidance tables commonly resume antiplatelets ∼24 h postop (agent-dependent)
Dual antiplatelet therapy	Evaluate indication (recent stent/ACS) and anaesthetic options; consider platelet function testing	Consider stopping 1 agent (typically the P2Y12 inhibitor) if clinically acceptable; otherwise proceed with general anaesthesia if neuraxial contraindicated	Specialist input recommended (cardiology/anaesthesia)	Typically 24–48 h postop for P2Y12 inhibitors in many periop protocols (context-dependent)
Vitamin K antagonist (VKA) (acenocoumarol, fenprocoumon, or warfarin)	INR target < 1.5 (often preferred for neuraxial)	Reverse on admission to avoid delay: give vitamin K 5 mg and recheck INR in 6–12 h; proceed once INR acceptable	If very urgent: consider PCC after discussion with haematology/blood bank (per pathway guidance). Bridging: LMWH pre-/postop only if high thrombotic risk (e.g. mechanical valve or recent VTE)/‘treatment VTE’ indication	24–36 h postop if haemostasis secure
DOACs – factor Xa inhibitors (apixaban, rivaroxaban, and edoxaban)	Consider DOAC level/LC-MS if available (not routine everywhere); renal function matters	Aim for early surgery; common approach is wait ∼24 h from last dose, extending to 24–48 h in moderate–severe renal impairment, if neuraxial planned or high bleed risk	Reversal generally reserved for life-threatening bleeding/high-risk situations; routine ‘reversal to operate’ is not standard. Bridging is not recommended during DOAC interruption in standard periop guidance	24–48 h postop if haemostasis secure
DOACs – thrombin inhibitor (dabigatran)	Consider assays if available; renal clearance is key	Typical approach: ∼24 h from last dose if renal function adequate; 24–48 h (or more) in impaired renal function/high bleed risk	Specific reversal exists (idarucizumab) but generally reserved for severe bleeding/critical need. Bridging not recommended in standard periop guidance	∼24 h postop if haemostasis secure

ACS, acute coronary syndrome; DOACs, direct oral anticoagulants.

### Anaemia

Anaemia is extremely common in patients presenting with a hip fracture and remains one of the most clinically relevant, potentially modifiable physiological derangements across the perioperative pathway. Studies consistently report a high prevalence of preoperative anaemia in geriatric hip fracture cohorts, often affecting roughly half of patients depending on the haemoglobin threshold used and case-mix, and many patients experience further postoperative haemoglobin decline (103, 104, 105, 106).

Anaemia has been associated with worse outcomes across multiple domains. First, it correlates with mortality and major complications, although causality is difficult to prove because anaemia also marks frailty and comorbidity. Still, several studies identify low haemoglobin as an independent predictor within prognostic models for hip fracture outcomes (104, 107, 108, 109, 110). Second, anaemia can impede functional recovery by limiting participation in early rehabilitation. Early mobilisation is a cornerstone of hip fracture recovery; when haemoglobin is low, patients may experience orthostatic symptoms, exertional intolerance, or cardiac strain, which can delay physiotherapy and increase the risk of deconditioning (111). Third, anaemia interacts with transfusion practice: patients with lower starting haemoglobin are more likely to receive transfusions, and transfusion itself has been associated in some settings with worse functional recovery, higher rates of infection, circulatory overload, and delirium, although confounding by illness severity is substantial (112, 113, 114, 115, 116, 117). The net effect is that anaemia can amplify vulnerability during the highest risk period after hip fracture – when cardiopulmonary complications, delirium, and immobility-related morbidity are most common.

Because transfusion is not benign and often does not improve functional outcomes beyond a restrictive threshold, attention has shifted to broader perioperative blood management strategies: (1) reducing bleeding, (2) treating reversible causes of anaemia, and (3) supporting erythropoiesis after surgery. Measures include careful anticoagulation management, meticulous surgical haemostasis, and the use of antifibrinolytics such as tranexamic acid (TXA) as part of institutional protocols (118, 119, 120). The FOCUS trial compared a liberal transfusion strategy (transfuse to maintain haemoglobin ≥ 10 g/dL) with a restrictive strategy (transfuse for symptoms of anaemia or haemoglobin < 8 g/dL) in high-risk patients after hip fracture surgery. The trial found that a liberal strategy did not improve survival or functional recovery compared with the restrictive strategy, supporting the safety of restrictive thresholds in this population (121). Subsequent practice-change analyses suggest that restrictive transfusion thresholds have been increasingly adopted after this evidence base matured (122).

### Infection

Intercurrent infection and systemic inflammation are common in older adults presenting with a hip fracture and are strongly linked to adverse outcomes. Many patients are frail, have multiple comorbidities, and have limited physiological reserve; as a result, even ‘mild’ infections (e.g. urinary tract infection (UTI)) or a heightened inflammatory state can precipitate delirium, decompensate cardiopulmonary disease, delay surgery, and increase mortality. The clinical challenge is to use infection/CRP information to improve outcomes without creating avoidable surgical delay, given the well-established harm associated with prolonged time to hip fracture surgery as noted above.

Large registry-based analyses demonstrate that infections such as pneumonia, UTI, and sepsis are common after a hip fracture and are influenced by baseline comorbidity and frailty; these infections contribute materially to morbidity and mortality during admission and after discharge (123). Preoperative infections matter because they can both reflect underlying vulnerability and trigger deterioration during the acute fracture episode. Preoperative pneumonia has been reported in 0.3–3.2% of hip fracture patients and may be a risk factor for adverse outcomes of hip fracture repair (124). Preoperative pneumonia has been identified as an independent predictor of 1-year mortality in geriatric hip fracture surgery cohorts, emphasising the high-risk phenotype of patients arriving with respiratory infection (125). Similarly, studies investigating UTI in the perioperative setting highlight that preoperative UTI is not rare and a pooled meta-analysis indicated an overall rate of 11% (95% CI: 0.08–0.14) (126). Preoperative UTI may increase the risk of early surgical site infection after hip fracture surgery (127), while broader analyses of UTI at the time of hip fracture admission explore impacts on outcomes and pathways of care (128).

The clinical goal is rapid, structured assessment and targeted management of infection/inflammation, avoiding indiscriminate delay. Evidence indicates that concurrent infection increases risk, but for many infections, the optimal approach is treat-and-proceed rather than postpone until complete resolution. In many patients with a UTI, starting antibiotics and proceeding to surgery once haemodynamically stable is reasonable. Delaying surgery purely for asymptomatic bacteriuria is generally not supported by strong evidence, while symptomatic UTI may warrant targeted therapy and monitoring (127, 128). Preoperative pneumonia is of higher risk and may require more active optimisation (oxygenation, bronchodilators, antibiotics, and fluid balance), but prolonged postponement can be harmful. Emphasis should be on stabilisation and early surgery when feasible, recognising that pneumonia itself predicts mortality (125).

CRP elevation is also common at admission and postoperatively. Admission CRP may rise due to (1) the fracture and associated tissue injury, (2) pre-existing infection or inflammatory disease, and/or (3) chronic inflammatory states (malnutrition, malignancy, and chronic organ disease). Postoperatively, CRP typically increases in response to surgical trauma and may follow predictable kinetics (129). Classic work describing postoperative CRP trajectories in hip fracture surgery suggests that deviations from expected patterns may help identify complications and infection (130, 131, 132, 133, 134, 135, 136). This is clinically useful because elderly hip fracture patients often have atypical infection presentations, and CRP trends can act as an additional signal when symptoms are subtle. Delaying surgery in patients with elevated serum-CRP levels suffering from femoral neck fractures may lead to worse survival and increased complication rates (137).

### Anaesthesia

Choice of anaesthetic technique is a key component of modern hip fracture pathways because it influences perioperative physiology, the ability to deliver timely surgery, early mobilisation, pain control, and complication profiles in a population that is typically elderly, frail, and comorbid. The two most common strategies are neuraxial anaesthesia (usually single-shot spinal anaesthesia, sometimes epidural or combined spinal–epidural) and general anaesthesia (GA), often delivered with multimodal adjuncts (opioids, hypnotics, volatile or total intravenous anaesthesia, and regional blocks).

Historically, neuraxial techniques were believed to confer some advantages – less thromboembolism, fewer pulmonary complications, and lower mortality – largely based on older trials and observational studies (138, 139, 140). However, contemporary high-quality randomised data and meta-analyses have challenged the magnitude and consistency of these differences and found little or no differences in outcome between the two techniques (141, 142, 143, 144, 145). Taken together, the literature supports a pragmatic conclusion: for many hip fracture patients, either spinal or general anaesthesia can be appropriate, and system factors such as availability, staff expertise, OR access and patient-specific contraindications may matter as much as the default technique.

Neuraxial anaesthesia may be preferred when airway risk is high, pulmonary reserve is limited, minimising systemic sedatives/opioids is desirable, and there is no contraindication (e.g. anticoagulation that cannot be safely managed; infection at the puncture site). General anaesthesia may be preferred when spinal placement is unlikely to succeed, urgent surgery is needed and neuraxial constraints would delay care, severe valvular stenosis or haemodynamic instability increases risk from sympathectomy, or patient factors (agitation; inability to cooperate) preclude safe neuraxial placement.

## Discussion

Hip fracture care has evolved from a primarily surgical focus towards recognition of the fracture as a systemic insult occurring in a highly vulnerable population. The consistent rise in absolute hip fracture numbers driven by population ageing, despite declining age-standardised incidence in many regions, underscores the growing importance of optimising perioperative pathways to mitigate morbidity and mortality. Early surgery remains a cornerstone of care, with extensive observational and randomised evidence demonstrating that unnecessary delays are associated with worse outcomes (18, 20, 21, 22). Rather than refining narrow time thresholds, contemporary emphasis should be placed on organisational models that prioritise rapid surgical access while correcting only those medical abnormalities that are reversible and clinically meaningful (Table 6).

**Table 6:** Key decision principles in the preoperative workup of hip fracture patients.

Should NOT delay surgery	Should prompt rapid treatment (not prolonged delay)
Mild anaemia	Active bleeding/symptomatic anaemia
Asymptomatic bacteriuria/CRP	Sepsis
Stable heart disease	Acute heart failure
Chronic lung disease	Hypoxia/pneumonia
Antiplatelet therapy	Uncontrolled anticoagulation
Mild electrolyte imbalance	Severe metabolic derangement

Preoperative optimisation is most effective when delivered through parallel multidisciplinary assessment rather than sequential medical clearance. Orthogeriatric co-management provides a structured framework to address frailty, multimorbidity, polypharmacy, cognitive vulnerability, and functional decline. Multiple trials and health-system evaluations demonstrate that such models reduce delirium, length of stay, complications, and mortality, reinforcing their central role in modern hip fracture pathways. Importantly, these benefits arise not from a single intervention but from coordinated bundles of care encompassing physiological stabilisation, pain management, delirium prevention, early mobilisation, and discharge planning (12, 13, 23, 25).

Pain control deserves particular emphasis as a modifiable driver of immobility, respiratory compromise, stress responses, and delirium. Multimodal systemic analgesia anchored by regular paracetamol and selective use of anti-inflammatory agents reduces opioid requirements and associated adverse effects. When combined with early regional techniques within structured protocols, analgesia improves substantially while facilitating nursing care, physiotherapy, and neuraxial anaesthesia positioning (41). However, emerging regional approaches such as the PENG block should be interpreted cautiously, as current evidence has not yet demonstrated clear superiority over established techniques.

Delirium prevention remains one of the most impactful targets for preoperative and perioperative intervention. Multicomponent non-pharmacological strategies addressing hydration, orientation, sleep, mobility, sensory input, and medication review consistently reduce delirium incidence and should be embedded into routine hip fracture care (56, 58, 59, 64). System-level multidisciplinary models further amplify these effects by ensuring early identification of high-risk patients and proactive management of precipitating factors.

Physiological derangements such as anaemia and intercurrent infection require pragmatic management aimed at stabilisation rather than prolonged postponement of surgery. Restrictive transfusion strategies, blood conservation measures, and targeted treatment of symptomatic infection align with contemporary evidence while minimising harm associated with delay (107, 121). Similarly, anaesthetic choice should be individualised, as modern randomised data suggest comparable outcomes between neuraxial and general techniques when delivered within optimised pathways (141).

## Conclusion

Overall, effective preoperative optimisation in hip fracture care is less about extensive investigation and more about rapid, coordinated intervention. Multidisciplinary systems that prioritise early surgery while addressing pain, delirium risk, physiological instability, and frailty represent the most evidence-aligned approach. Future research should focus on refining care bundles, improving implementation fidelity across healthcare systems, and identifying patient-specific strategies that further reduce complications and improve long-term functional outcomes.

## ICMJE Statement of Interest

The authors declare that there is no conflict of interest that could be perceived as prejudicing the impartiality of the work reported.

## Funding Statement

No benefits in any form have been received or will be received from a commercial party related directly or indirectly to the subject of this article.
